# Selective Serotonin Reuptake Inhibitor Use and Risk of Major Bleeding during Treatment with Vitamin K Antagonists: Results of A Cohort Study

**DOI:** 10.1055/a-1957-6305

**Published:** 2022-12-30

**Authors:** Sanne Bakker, Johanna Louise I. Burggraaf, Marieke J. H. A. Kruip, Felix J. M. van der Meer, Willem M. Lijfering, Nienke van Rein

**Affiliations:** 1 Department of Clinical Pharmacy and Toxicology, Leiden University Medical Center, Leiden, The Netherlands; 2 Department of Clinical Epidemiology, Leiden University Medical Center, Leiden, The Netherlands; 3 Department of Hematology, Erasmus University Medical Center, Rotterdam, The Netherlands; 4 Star-shl Thrombosis Service, Rotterdam, The Netherlands; 5 Department of Thrombosis and Hemostasis, Leiden University Medical Center, Leiden, The Netherlands

**Keywords:** SSRI, serotonin reuptake inhibitors, cohort study, drug interactions, hemorrhages, coumarins

## Abstract

**Background** Selective serotonin reuptake inhibitors (SSRIs) may increase the risk of major bleeding by decreasing platelet function or decreasing vitamin K antagonist (VKA) metabolism via cytochrome P450 (CYP) inhibition.

**Aims** To determine whether SSRIs are associated with major bleeding during VKA treatment and investigate the possible mechanisms.

**Methods** In this cohort study, information on SSRI use and bleeding complications was obtained from patient records of VKA initiators between 2006 and 2018 from two anticoagulation clinics. Conditional logistic regression and time-dependent Cox regression were used to estimate the effect of SSRIs on a high international normalized ratio (INR ≥ 5) within 2 months after SSRI initiation and on major bleeding during the entire period of SSRI use, respectively. SSRI use was stratified for (non-)CYP2C9 inhibitors.

**Results** A total of 58,918 patients were included, of whom 1,504 were SSRI users. SSRI initiation versus nonuse was associated with a 2.41-fold (95% confidence interval [CI]: 2.01–2.89) increased risk for a high INR, which was 3.14-fold (95% CI: 1.33–7.43) among CYP2C9-inhibiting SSRI users. The adjusted hazard ratio of major bleeding was 1.22 (95% CI: 0.99–1.50) in all SSRI users and 1.31 (95% CI: 0.62–2.72) in CYP2C9-inhibiting SSRI users compared with nonusers.

**Conclusion** SSRI use is associated with an increased risk of high INR and might be associated with major bleeding. The risk of a high INR was slightly more elevated for CYP2C9-inhibiting SSRI users, suggesting there might be a pharmacokinetic interaction (by CYP2C9 inhibition) next to a pharmacodynamic effect of SSRIs on platelet activation.

## Graphical abstract

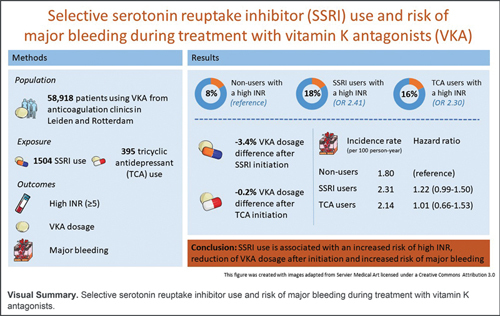

## Introduction

Vitamin K antagonists (VKAs) are used to treat and prevent thrombosis. In the Netherlands, patients using VKAs are monitored by anticoagulation clinics, which regularly measure the international normalized ratio (INR). Despite this intensive monitoring system, 1 to 3% of the VKA users suffer from major bleeding each year. 1 2 In addition to the effect of VKAs, co-medication such as selective serotonin reuptake inhibitors (SSRIs) may be associated with an increased risk of bleeding. 3 4 5 6 7 There are three possible causal mechanisms by which SSRIs could increase the risk of bleeding described in previous literature. First, SSRIs inhibit serotonin reuptake in platelets which might result in a decreased platelet function leading to an increased risk of bleeding. 8 Second, SSRIs might increase gastric acid secretion, which might increase the risk of upper gastrointestinal bleeding by increasing the risk of peptic ulcer disease. 9 Third, the SSRIs fluvoxamine and fluoxetine have a pharmacokinetic interaction with VKAs through inhibition of cytochrome P450 2C9 (CYP2C9), which is the most important enzyme for the metabolism of VKAs. 10 11 This inhibition could lead to a high INR and consequently an increased bleeding risk. Previous studies showed that use of fluvoxamine (a CYP2C9 inhibitor) is associated with an increased risk of a high INR compared with nonusers of SSRIs, 12 but have not studied major bleeding as an outcome. Other studies reported relative risk estimates of major bleeding between 1.0 and 1.7, but were underpowered as shown by large confidence intervals (CIs) around these point estimates and correction for potential confounders was not optimal. 4 6 7 Furthermore, none of these studies included phenprocoumon users, while phenprocoumon is metabolized by both CYP2C9 and CYP3A4, whereas acenocoumarol and warfarin are predominantly metabolized by CYP2C9. These differences in metabolism may make phenprocoumon less prone to the pharmacokinetic interaction with SSRIs through CYP2C9 inhibition as compared with acenocoumarol and warfarin. 13

Because of these limitations, there is still uncertainty regarding the increased risk of major bleeding due to SSRI use in patients treated with a VKA and possible mechanisms of these bleeds have not been studied extensively. To offer patients the most optimal treatment for both depression and thrombosis, these mechanisms may provide clues to what antidepressants are preferred in patients using VKAs and which type of VKA might be preferred in patients using SSRI. We therefore determined whether SSRIs are associated with major bleeding during VKA treatment and investigated possible mechanisms that might cause major bleeding. To establish this aim, we performed a cohort study, in which both the INR and major bleeding were studied as outcomes.

## Methods

### Study Population and Data Collection

In this cohort study, all patients aged 18 years or older who started using a VKA between January 1, 2006 and January 1, 2018 at the Anticoagulation Clinic Leiden or the Thrombosis Service of Star-shl, Rotterdam, were included. They were followed from the start of VKA use until they stopped using the VKA, moved to a city not covered by the anticoagulation clinics, died, or reached the end of the study period (January 2018).

Patient characteristics, exposures, and outcomes were derived from the electronic patient records of both anticoagulation clinics. 14 There, the INR of every patient is measured every 1 to 6 weeks; the interval between INR measurements is based on the stability of the INR and the previous INR measurement. At every monitoring visit, nurses take a standardized short questionnaire including questions on changes of co-medication and the occurrence of bleeds. Bleeding events are reported to the anticoagulation clinic by physicians and re-enlisting forms to the anticoagulation clinic after a hospital discharge. 15 Records of the cohort include age, sex, indication for VKA therapy, type of VKA (acenocoumarol or phenprocoumon), INR target range, INR measurements, the occurrence of bleedings, VKA dosage, and concomitant medication use (such as SSRIs). 16 Institutional review board approval was obtained through Medical Ethical Committee of the Leiden University Medical Centre; participant consent was waived because the analysis used preexisting, coded data.

### Exposure and Outcomes

The starting date and end date of SSRI use were reported by the patient or the drug-dispensing pharmacy. Patients using SSRI at baseline were classified as prevalent users; they were considered exposed to SSRI from baseline. Patients who initiated a SSRI during the study period were classified as incident users and considered exposed from the start date of the SSRI. The exposure period ended 30 days after the date SSRI use was discontinued during follow-up or when the end of the study period was reached. We added these extra 30 days to the exposure period to account for the elimination of the SSRI. SSRI use was stratified by CYP2C9-inhibiting SSRIs.

The three studied outcomes were high INR, difference in VKA dosage, and major bleeding, which are described below. A high INR was defined as an INR above 4 or above 5. Both cut-offs were considered since an INR above 4 is associated with a slight increased risk of major bleeding and an INR above 5 is associated with an apparent increased risk of major bleeding. 17 For the analysis with high INR as an outcome, we matched every VKA user who initiated treatment with SSRIs with up to five non-SSRI users on age, sex, type of VKA, duration of VKA treatment, VKA indication, and use of proton-pump inhibitors (PPIs), nonsteroidal anti-inflammatory drugs (NSAIDs) and antiplatelet drugs. We matched on the duration of VKA use, since INR values outside the therapeutic range are more common in the first period after the initiation of VKA. When a SSRI was initiated within 30 days of VKA initiation, the matching nonusers used VKA for a maximum of 15 days longer. A maximum difference of 30 days was used, when the SSRI was initiated between day 30 and day 360 after VKA initiation. When a SSRI was initiated after day 360 of VKA use, a difference in the duration of VKA use with the matching nonusers of maximum 180 days was accepted. The presence of a high INR was considered during a period of 2 months after SSRI initiation for the SSRI initiators or match day for the matched non-SSRI users.

To verify the results of the analysis with high INR as an outcome, we included VKA dosage as an outcome. If a pharmacokinetic interaction occurs between VKA and SSRI, we expected that a high INR would occur, after which the dosage of VKAs would decrease. Therefore, for SSRI users the difference in VKA dosage before the start of the SSRI and 2 months after the start of the SSRI was also calculated. Dosage changes were considered in tablets (1 tablet acenocoumarol equals 1 mg and 1 tablet phenprocoumon 3 mg) and in percentages with the dosage before SSRI initiation as the reference category. Both measures were considered since relative dosage changes (percentages) are more comparable when the amounts of tablets differ at baseline, but dosage change in tablets might be more intuitive for VKA prescribers.

Major bleeding was defined as fatal bleeding, bleeding in a critical area or organ, or bleeding resulting in hospitalization or blood transfusion and classified according to the location of bleeding (i.e., intracranial bleeding, gastrointestinal bleeding, ocular bleeding, cutaneous bleeding, joint and muscular bleeding, urogenital bleeding, respiratory tract bleeding, traumatic bleeding, and other types of major bleeding). We stratified the analysis for site of bleeding to explore whether there was a marked increase in the risk of gastrointestinal bleeding which might point to the mechanism of increased gastric acid secretion.

### Statistical Analyses

For the analyses with major bleeding as an outcome, we used a Cox proportional hazards model with time-dependent covariates to estimate crude and adjusted hazard ratios (HRs) and 95% CIs. We adjusted for sex and the time-varying confounders age, indication of VKA treatment, INR target range, PPIs, NSAIDs, and antiplatelet drugs in the analysis. For this analysis, patients were followed until the first occurrence of a major bleeding and censored at the moment they stopped using the VKA, moved to a city not covered by the anticoagulation clinics, died, or reached the end of the study period (January 2018).

To study the association of SSRI use with a high INR, we estimated odds ratios with 95% CIs by means of conditional logistic regression. Furthermore, the association of SSRI use with VKA dosage was studied by performing a paired *t* -test.

In all analyses, SSRI users were stratified by CYP2C9-inhibiting SSRIs to be able to study a potential pharmacokinetic interaction. In addition, the results were stratified by type of VKA to explore a potential difference between phenprocoumon and acenocoumarol. To account for confounding by indication (i.e., the indication depression leads to the outcome of a high INR or major bleeding), we performed a sensitivity analysis in tricyclic antidepressant (TCA) users as a negative control. 18 Since mirtazapine and nortriptyline are commonly used antidepressants and have, just like maprotiline and doxepin, the lowest affinity for the serotonin transporter, these four TCAs were chosen. 19 Patients using both SSRI and TCA at the same time were classified as SSRI users. A second sensitivity analysis was conducted for the analysis on major bleeding to rule out prevalent user bias, 16 in which we stratified the analysis based on incident and prevalent SSRI users.

All analyses were performed with R, version 4.1.0, a language and environment for statistical computing and graphics.

## Results

A total of 58,918 patients started VKA therapy within the study period. Of these, 57,019 were nonusers, 1,504 were SSRI users, and 395 were TCA users at baseline. The number of treatment periods was higher than the number of patients, since some patients were treated with a VKA multiple times and discontinued treatment in between. This resulted in 61,245 treatment periods for baseline nonusers, 1,557 treatment periods for baseline SSRI users, and 404 treatment periods for baseline TCA users. Of all patients at baseline, 52% ( *n* = 32,898) were male, while in the groups of SSRI users and TCA users, 31% ( *n* = 488) and 39% ( *n* = 159) patients were male, respectively. At baseline, the mean age was 69 years (standard deviation: 15). The most common indications for use of a VKA were atrial fibrillation (56%) and venous thromboembolism (27%), and a low INR target range was used in most treatment periods (91%). Acenocoumarol was used by 97% ( *n* = 41,394) of the patients at the anticoagulation clinic in Rotterdam as compared with 15% ( *n* = 3,016) of the patients at the anticoagulation clinic in Leiden. Concerning co-medication, users of a SSRI or a TCA used a NSAID twice as often (6 and 5%, respectively) compared with nonusers (3%). The prevalence of PPI use was higher among SSRI (44%) and TCA users (38%) compared with nonusers (15%) ( Table 1 ). A total of 1,182 patients initiated SSRI and 456 patients initiated TCA during the study period. At the time of initiation of SSRI and TCA for the initiators, the mean age at initiation was 76 and 79 years, respectively ( Supplementary Table S1 [online only] for all initiators, Supplementary Table S2 for initiators included in the major bleeding analysis [online only]).

**Table 1:** Baseline characteristics of the entire cohort (at the start of VKA use)

General characteristics	Nonusers	SSRI users	TCA users
Patients	57,019	1,504	395
Treatment periods	61,245	1,557	404
Male (%)	32,251 (53)	488 (31)	159 (39)
Age, y (SD)	69 (15)	69 (15)	74 (14)
INR target range (%)			
Low	55,676 (91)	1,454 (93)	378 (94)
High	5,569 (9)	103 (7)	26 (6)
Treatment indication (%)			
Atrial fibrillation	34,498 (56)	800 (51)	245 (61)
Venous thromboembolism	16,642 (27)	534 (34)	107 (27)
Mechanical heart valves	2,892 (5)	67 (4)	9 (2)
Vascular disease	2,474 (4)	57 (4)	21 (5)
Ischemic heart disease	2,528 (4)	51 (3)	17 (4)
Postoperative	1,561 (3)	24 (2)	6 (2)
Other	856 (1)	30 (2)	3 (1)
Vitamin K antagonist (%)			
Phenprocoumon	17,874 (29)	672 (43)	146 (36)
Acenocoumarol	43,269 (71)	883 (57)	258 (64)
Warfarin	97 (0)	2 (0)	0 (0)
Fluindione	5 (0)	0 (0)	0 (0)
Anticoagulation clinic (%)			
Leiden	19,646 (32)	738 (47)	175 (43)
Rotterdam	41,599 (68)	819 (53)	229 (57)
Co-medication (%)			
Antiplatelet drugs	5,222 (9)	202 (13)	59 (15)
NSAIDs	1,531 (3)	99 (6)	18 (5)
Proton pump inhibitors	9,435 (15)	681 (44)	153 (38)

Abbreviations: SSRI, selective serotonin reuptake inhibitor; TCA, tricyclic antidepressant.

### Risk of High INR after SSRI or TCA Initiation

Of the 1,175 included SSRI initiators included in this analysis, 211 patients (18%) had a high INR (INR ≥ 5) at least once in the first 2 months after SSRI initiation, compared with 474 out of the 5,637 matched nonexposed patients (8%) with at least one high INR in the 2 months after the matching date. SSRI initiation was associated with a 2.41-fold (95% CI: 2.01–2.89) increased risk for a high INR compared with matched nonusers ( Table 2 ). After stratification for CYP2C9-inhibiting SSRIs, the relative risk of a high INR after initiation of treatment among CYP2C9-inhibiting SSRI users was 3.14 (95% CI: 1.33–7.43), compared with matched nonusers. The relative risk of a high INR for users of the other SSRIs was 2.38 (95% CI: 1.98–2.86), compared with matched nonusers. TCA initiation was associated with a 2.30-fold (95% CI: 1.70–3.12) increased risk for a high INR compared with matched non-TCA users ( Table 2 ). The analyses with an INR ≥4 as an outcome showed similar patterns compared with the analysis with INR ≥5 as an outcome ( Supplementary Table S3 [online only]).

**Table 2:** Association of SSRI and TCA initiation with a high INR (≥5) within 2 months after initiation stratified by VKA type

	Total no. of patients	No. of patients with at least 1 INR ≥ 5 (%)	OR (95% CI) INR ≥ 5
All patients			
Matched non-SSRI users	5,637	474 (8)	Reference
All SSRI users	1,175 a	211 (18)	2.41 (2.01–2.89)
CYP2C9-inhibiting SSRIs	62	10 (16)	3.14 (1.33–7.43)
Non-CYP2C9-inhibiting SSRIs	1,113	201 (18)	2.38 (1.98–2.86)
Matched non-TCA users	2,195	163 (7)	Reference
TCA users	451 a	70 (16)	2.30 (1.70–3.12)
Acenocoumarol patients			
Matched non-SSRI users	3,341	330 (10)	Reference
All SSRI users	689	149 (22)	2.52 (2.03–3.13)
CYP2C9-inhibiting SSRIs	37	7 (19)	2.94 (1.07–9.04)
Non-CYP2C9-inhibiting SSRIs	652	142 (22)	2.50 (2.00–3.13)
Matched non-TCA users	1,537	119 (8)	Reference
TCA users	313	59 (19)	2.78 (1.97–3.91)
Phenprocoumon patients			
Matched non-SSRI users	2,293	144 (6)	Reference
All SSRI users	484	61 (13)	2.17 (1.58–2.99)
CYP2C9-inhibiting SSRIs	25	3 (12)	3.78 (0.72–19.88)
Non-CYP2C9-inhibiting SSRIs	459	58 (13)	2.13 (1.53–2.95)
Matched non-TCA users	658	44 (7)	Reference
TCA users	138	11 (8)	1.19 (0.59–2.38)

Abbreviations: CYP2C9, cytochrome P450 2C9; SSRI, selective serotonin reuptake inhibitor; TCA, tricyclic antidepressant; VKA, vitamin K antagonist.

a Twelve SSRI initiators and 5 TCA initiators were not included in this analysis because no suitable match could be found.

### Dosage Change after Initiating a SSRI or TCA

The mean difference in dosage after SSRI initiation was −3.4% (95% CI: −4.5 to −2.3). A dosage difference of −8.6% (95% CI: −14.2 to −2.9) was observed when restricting to CYP2C9-inhibiting SSRIs. After stratifying for VKAs, the mean dosage difference of acenocoumarol was −2.9% (95% CI: −4.5 to −1.2) and for phenprocoumon −4.1% (95% CI: −5.3 to −2.8). For TCA initiators the mean dosage difference was around zero. Similar results were found when considering tablet dosage change ( Supplementary Table S4 [online only]).

### Risk of Major Bleeding during SSRI or TCA Use

During a follow-up of 137,407 patient-years, 2,504 major bleedings occurred. The most common site of bleeding was gastrointestinal bleeding, followed by intracranial bleeding. SSRI users had an incidence rate for major bleeding of 2.31/100 person-years (95% CI: 1.88–2.80) and nonusers had an incidence rate of 1.80/100 person-years (95% CI: 1.73–1.88). Incidence rates were similar for CYP2C9-inhibiting SSRIs and the non-CYP2C9-inhibiting SSRIs across all subgroups.

The crude HR of major bleeding in SSRI users was 1.31 (95% CI: 1.07–1.61), compared with nonusers, which was 1.22 (95% CI: 0.99–1.50) after adjustment for confounding. After stratification for CYP2C9-inhibiting SSRIs, adjusted risk estimates remained similar (CYP2C9-inhibiting SSRIs: HR: 1.30, 95% CI: 0.62–2.72 vs. non-CYP2C9 inhibiting SSRIs: HR: 1.21, 95% CI: 0.98–1.50). Among acenocoumarol users, the HR of major bleeding in SSRI users was 1.26 (95% CI: 0.95–1.68), compared with nonusers, and among phenprocoumon users the risk of major bleeding for SSRI users compared with nonusers was around unity (HR: 1.05, 95% CI: 0.78–1.41). The risk estimates of major bleeding for TCA users as compared with nonusers were around unity (HR: 1.01, 95% CI: 0.66–1.53) (see Table 3 and Supplementary Table S5 for crude HRs [online only]).

**Table 3:** Association of SSRI use with major bleeding stratified by VKA type

	Person-years	No. of cases with major bleeding	Incidence rate per 100 person-years (95% CI)	HR a (95% CI)
All patients				
Nonusers	132,176	2,385 b	1.80 (1.73–1.88)	Reference
All SSRI users	4,204	97	2.31 (1.88–2.80)	1.22 (0.99–1.50)
CYP2C9-inhibiting SSRIs	321	7	2.18 (0.95–4.31)	1.30 (0.62–2.72)
Non-CYP2C9-inhibiting SSRIs	3,883	90	2.32 (1.88–2.84)	1.21 (0.98–1.50)
TCA users	1,026	22	2.14 (1.38–3.19)	1.01 (0.66–1.53)
Acenocoumarol patients				
Nonusers	100,138	1,546	1.54 (1.47–1.62)	Reference
All SSRI users	2,604	49	1.88 (1.41–2.47)	1.26 (0.95–1.68)
CYP2C9-inhibiting SSRIs	201	4	1.99 (0.63–4.80)	1.46 (0.55–3.91)
Non-CYP2C9-inhibiting SSRIs	2,403	45	1.87 (1.38–2.48)	1.24 (0.92–1.68)
TCA users	676	13	1.92 (1.07–3.21)	1.10 (0.64–1.90)
Phenprocoumon patients				
Nonusers	31,875	838	2.63 (2.46–2.81)	Reference
All SSRI users	1,595	48	3.01 (2.24–3.96)	1.05 (0.78–1.41)
CYP2C9-inhibiting SSRIs	120	3	2.50 (0.64–6.80)	1.04 (0.33–3.23)
Non-CYP2C9-inhibiting SSRIs	1,475	45	3.05 (2.25–4.05)	1.05 (0.78–1.43)
TCA users	350	9	2.57 (1.25–4.72)	0.82 (0.43–1.59)

Abbreviations: CI, confidence interval; HR, hazard ratio; CYP2C9, cytochrome P450 2C9; SSRI, selective serotonin reuptake inhibitor; TCA, tricyclic antidepressant; VKA, vitamin K antagonist.

a Time-dependent analysis adjusted for sex and time-dependent covariates age, INR target range, indication for VKA, and co-medication (antiplatelet drugs, NSAIDs, and PPIs).

b One of these major bleedings was observed in a warfarin user.

The risk estimate for gastrointestinal bleeding in SSRI users was similar to the overall risk of bleeding in SSRI users ( Supplementary Table S6 [online only]).

### Sensitivity Analysis: Incidence of Major Bleeding in Incident versus Prevalent Users

The analysis on incident and prevalent SSRI users showed a HR of 1.15 (95% CI: 0.82–1.62) for major bleeding in incident SSRI users (initiating a SSRI) and a HR of 1.26 (95% CI: 0.98–1.63) for prevalent users (use at baseline) compared with nonusers. When stratifying for VKAs, phenprocoumon users showed similar results. In contrast, among acenocoumarol users we observed a HR for major bleeding of 1.45 (95% CI: 0.95–2.22) for incident SSRI users, while this was 1.14 (95% CI: 0.78–1.67) for prevalent users as compared with nonusers ( Table 4 and Supplementary Table S7 for crude HRs [online only]).

**Table 4:** Association of SSRI use with major bleeding for incident and prevalent users stratified by VKA type

	Person-years	No. of major bleedings	Incidence rate per 100 person-years (95% CI)	HR a (95% CI)
All patients				
Nonusers	132,176	2,385 b	1.80 (1.73–1.88)	Reference
Incident SSRI users	1,837	35	1.91 (1.35–2.62)	1.15 (0.82–1.62)
Prevalent SSRI users	2,367	62	2.62 (2.03–3.34)	1.26 (0.98–1.63)
Incident TCA users	528	12	2.27 (1.23–3.86)	1.22 (0.69–2.16)
Prevalent TCA users	498	10	2.01 (1.02–3.56)	0.83 (0.45–1.55)
Acenocoumarol patients				
Nonusers	100,138	1,546	1.54 (1.47–1.62)	Reference
Incident SSRI users	1,157	22	1.90 (1.22–2.83)	1.45 (0.95–2.22)
Prevalent SSRI users	1,447	27	1.87 (1.26–2.68)	1.14 (0.78–1.67)
Incident TCA users	329	8	2.43 (1.13–4.62)	1.57 (0.78–3.16)
Prevalent TCA users	348	5	1.44 (0.53–3.19)	0.73 (0.31–1.78)
Phenprocoumon patients				
Nonusers	31,875	838	2.63 (2.46–2.81)	Reference
Incident SSRI users	677	13	1.92 (1.07–3.20)	0.74 (0.43–1.28)
Prevalent SSRI users	918	35	3.81 (2.70–5.24)	1.26 (0.89–1.77)
Incident TCA users	199	4	2.01 (0.64–4.85)	0.72 (0.27–1.93)
Prevalent TCA users	151	5	3.31 (1.21–7.34)	0.93 (0.39–2.25)

Abbreviations: CI, confidence interval; HR, hazard ratio; SSRI, selective serotonin reuptake inhibitor; TCA, tricyclic antidepressant; VKA, vitamin K antagonist.

a Time-dependent analysis adjusted for sex and time-dependent covariates age, INR target range, indication for VKA, and co-medication (antiplatelet drugs, NSAIDs, and PPIs).

b One of these major bleedings was observed in a warfarin user.

## Discussion

This study shows that the initiation of a SSRI during VKA treatment is associated with an increased risk of a high INR during the first 2 months after initiation. For all SSRI initiators, the risk was twofold increased, and when restricting to CYP2C9-inhibiting SSRIs, the initiation was associated with a threefold increased risk. Initiation of non-CYP2C9-inhibiting SSRIs or TCAs was associated with a twofold increased risk of a high INR. Furthermore, we observed the risk of major bleeding for concomitant users of VKAs and all SSRIs is possibly slightly increased. When stratified for CYP2C9-inhibiting SSRIs and the non-CYP2C9-inhibiting SSRIs, similar results were observed for both groups. TCA users did not show increased risk estimates of major bleeding.

### SSRI Use and a High INR and VKA Dosage

We found an increased risk of a high INR and a corresponding decrease in VKA dosage in all SSRI initiators compared with nonusers, which was in line with a previous study. 12 Beforehand, we expected this only for the CYP2C9-inhibiting SSRIs, due to the pharmacokinetic interaction. 10 11 When restricting to CYP2C9-inhibiting SSRIs, a higher risk of an increased INR and a stronger dosage decrease were observed compared with non-CYP2C9-inhibiting SSRIs, which might indicate that CYP interaction decreases the metabolism of VKAs, resulting in a higher INR (i.e., the pharmacokinetic mechanism), but a chance finding cannot be excluded.

For TCA initiators the risk of a high INR was increased, but the mean dosage difference was around zero. These results seem contradictory, however, VKA dosage can be influenced by other factors. Since the risk of a high INR was also increased for TCA initiators (our negative control), the increased risk is possibly partly explained by confounding due to the indication depression. Depressed patients might take less care of themselves and eat less, including green vegetables containing vitamin K, which may result in increased occurrence of high INR. This hypothesis is supported by multiple studies that showed that patients suffering from a mental illness had a lower time in INR target range than healthy VKA users. 20 21 22

### SSRI Use and Major Bleeding

We observed a possible association between SSRI use and major bleeding in patients using VKA, with a small increased risk of major bleeding for SSRI users. To interpret this result, several aspects must be considered. Although a chance finding cannot be excluded entirely since the lower limit of the 95% CI for major bleeding in SSRI users is just below 1.0, the observed risk estimate for major bleeding is confirmed by the results of previous research showing an increased risk for major bleeding in SSRI, 4 6 making a chance finding less likely. Furthermore, as in all observational studies, residual confounding should be considered. However, our sensitivity analysis showed that TCA use was not associated with an increased risk of major bleeding compared with nonusers, which corresponds with results from previous research 5 and suggests absence of confounding by indication (i.e., depression) for the outcome of major bleeding. Therefore, it is possible that SSRI use is associated with an increased risk of major bleeding, which supports the hypothesis of platelet inhibition.

Although the risk estimate of major bleeding for patients using CYP2C9-inhibiting SSRIs was slightly more pronounced compared with non-CYP2C9-inhibiting SSRI users, the number of events in the patients using CYP2C9-inhibiting SSRIs is too low to draw formal conclusions regarding CYP interaction and the association with major bleeding.

After stratification for type of VKA, no association was found between SSRI use and major bleeding in phenprocoumon users, while for acenocoumarol users we observed the risk of major bleeding among the SSRI users was possibly elevated. This difference could be due to the long half-life of phenprocoumon, ensuring more stable INRs 23 24 and potentially fewer major bleedings. Furthermore, the inhibition of CYP2C9 is expected to influence the metabolism of phenprocoumon less than acenocoumarol, because the enzyme CYP3A4 is also involved in the metabolism of phenprocoumon. 1 13 25 26 This may explain why no difference in risk estimates was observed between CYP2C9-inhibiting SSRIs and the non-CYP2C9-inhibiting SSRIs in phenprocoumon users and may support that the pharmacokinetic interaction may explain the increased risk for major bleeding among SSRI users. However, the number of events in the analysis stratified for type of VKA is too low to draw formal conclusions.

The analysis stratified for site of bleeding showed that the risk of gastrointestinal bleeding for SSRI users might possibly be increased compared with nonusers, which is in line with previous research. 27 28 In case SSRIs increase gastric secretion, we would have expected that the risk of gastrointestinal bleeding would be increased more, compared with other locations. However, the risks for gastrointestinal major bleeding and intracranial bleeding and cutaneous major bleeding were similar. Therefore, our results did not indicate that major bleedings are caused by increased gastric acid secretion by SSRIs. A possible explanation may be the fact that SSRIs increase gastric acid secreting has only been studied in vitro and in rodent models, 29 30 so it remains unclear to what extend this mechanism is relevant for the risk of bleeding in patients using a SSRI. However, a limitation of our data was that details regarding the location (i.e., upper or lower) of gastrointestinal bleeding were not available. Therefore, we were not able to estimate the risk for upper gastrointestinal bleeding separately, and it is possible we missed a potential association.

### Prevalent User Bias

Due to prevalent user bias, we expected beforehand that we would find higher relative risk estimates of major bleeding among incident users than prevalent users. Incident users are more representative for the population who start using a drug (here SSRIs). Prevalent users on the other hand are expected to be a selected group of patients who are relatively healthier, since they are physically able to continue using the SSRI and do not stop using the SSRI early, caused by for example side effects. In addition, they have a higher compliance to drugs and suffer less from side effects such as major bleedings. 16 Surprisingly, in prevalent SSRI users a slightly higher risk estimate of major bleeding was observed than in incident SSRI users. A possible explanation for this result could be that longer use of a SSRI may indicate that these patients have a more severe depression. Depression is associated with conditions such as coronary heart disease, diabetes, hypertension, and atrial fibrillation, 3 which are also risk factors for major bleeding. 2 As mentioned before, the shorter half-life of acenocoumarol compared with phenprocoumon could result in less stability in INR 23 24 and consequently an increased risk of major bleeding when starting a new drug, which is supported by our results. Another explanation is that the difference can be caused by small numbers and statistical variation.

### Strengths and Limitations

More than 58,000 patients were included in our study, which is a strength as robust risk estimates were found compared with other studies. Furthermore, we have included TCA users as the negative control to detect potential confounding by indication (i.e., depression). Another strength is that we included phenprocoumon users in our study, which has a different pharmacokinetic profile from acenocoumarol, and gave us the opportunity to further study the mechanism by which SSRIs may cause major bleeding. Next to the limitations already mentioned above, our study has the following additional limitations. First, the use of co-medication, under which SSRIs and TCAs fall, is reported by the patients or drug-dispensing pharmacies in case of interacting co-medication (such as the CYP2C9-inhibiting SSRIs). This could have resulted in underreporting of SSRI and TCA use and thereby misclassification of the exposure status, which would result in underestimation of the observed risk estimates. Second, the bleeding events were reported by patients or physicians, which might result in underreporting. However, we do not expect this to be dependent on the exposure status. Therefore, this might result in underestimation of the incidence rates of major bleeding, but we do not expect this to influence our relative risk estimates. Third, information was not available on the indication and dosages of SSRIs and TCAs and adjustment for the indication depression was not possible. Therefore, we included TCA as a negative control to detect confounding by depression. However, SSRIs are also used for the indication anxiety, and TCAs in a low dosage also against neuropathic pain. This may have led to suboptimal detection of confounding by indication. However, it is likely though that most included patients used a SSRI or a TCA against depression. Fourth, our cohort consists of patients who started using a VKA between 2006 and 2018. During this period direct oral anticoagulants (DOACs) were introduced and since 2016 the majority of patients who initiated anticoagulant therapy were prescribed a DOAC. 31 Since nowadays DOACs are the first-choice anticoagulant for most indications, characteristics of current VKA users might be different compared with our study population, including elderly patients who started VKA therapy before the DOAC era as well as patients with contraindication for DOACs such as patients with renal insufficiency. Since these characteristics are associated with major bleeding, the observed incidence of major bleeding might be an underestimation of the current risk of major bleeding. Last, despite the large total number of included patients, some subgroups, especially the CYP2C9-inhibiting SSRI users, had a small sample size, which limited the statistical power and resulted in wide confidence intervals.

### Clinical Implications

To take our results into account in clinical practice, several aspects must be considered. The risk of a high INR for the non-CYP2C9-inhibiting SSRI initiators or users was lower than that for CYP2C9-inhibiting SSRIs compared with nonusers and the dosage decrease was weaker. Therefore, when a patient has to start a SSRI when already using a VKA, we would suggest starting a non-CYP2C9-inhibiting SSRI, based on our results. Since the indication depression might play a role, it could be useful to have more intensive monitoring during the first 2 months of SSRI and TCA use.

Next to VKA, DOACs are frequently used to treat and prevent thrombosis nowadays. This might raise the question of whether our results apply to DOAC users or whether DOACs and SSRI also interact. CYP metabolism has an important role in the elimination of apixaban and rivaroxaban, but occurs mainly by CYP3A4. 32 Since CYP2C9 does not play a major role in the metabolism of DOACs, we do not expect a relevant pharmacokinetic interaction on CYP level between SSRI and DOACs. However, in contrast to VKA, DOACs are substrates of the P-glycoprotein (P-gp) transporter, a transport protein on the cell membrane which is expressed in the gut, liver, and kidneys, that has a role in pharmacokinetics. Paroxetine, fluvoxamine, and sertraline might inhibit P-gp activity and thereby might cause a pharmacokinetic interaction with DOACs. 33 Next to this, the pharmacodynamic effect of SSRIs on platelets is expected to be present in DOAC users as well. Therefore, users of both DOACs and SSRIs might be at increased risk of major bleeding. Indeed, Zhang et al reported a 1.68-fold (95% CI: 1.10–2.59) increased risk of major bleeding in SSRI users as compared with nonusers in a cohort with incident DOAC users. 34 However, the analysis was not stratified for different types of SSRIs, therefore it is not known whether a certain type of SSRI should be preferred with regard to the risk of major bleeding. As far as we are aware, the risks of major bleeding for SSRI users in DOAC versus VKA users have not been studied.

## Conclusion

In conclusion, SSRI initiation and in particular the initiation of CYP2C9-inhibiting SSRIs is associated with an increased risk of a high INR during VKA use, which could result in major bleeding. Based on our results, an association between SSRI use and increased risk of major bleeding during VKA use is highly possible. These results suggest that platelet inhibition and a pharmacokinetic interaction (by CYP2C9 inhibition) both may play a role in the occurrence of a high INR and major bleedings after SSRI initiation and during SSRI use.

## Notes

**What is known about this topic?**

- Selective serotonin reuptake inhibitors (SSRIs) may increase the risk of major bleeding by decreasing platelet function or interaction with vitamin K antagonist (VKA) metabolism via cytochrome P450 (CYP).

**What does this paper add?**

- SSRI use was associated with an increased risk of a high international normalized ratio (INR) and might be associated with major bleeding.
- The risk of a high INR was slightly more increased after initiation of a CYP2C9-inhibiting SSRI, supporting the hypothesis of CYP interaction.
- We recommend using a non-CYP-inhibiting SSRI and a close INR monitoring.
