# Supplementary material for: Selective Serotonin Reuptake Inhibitor Use and Risk of Major Bleeding during Treatment with Vitamin K Antagonists: Results of A Cohort Study
Source: Thromb Haemost. 2022 Dec 30;123(2):245–54. doi: 10.1055/a-1957-6305 (PMC9904966; doi:10.1055/a-1957-6305)
Supplement: Supplementary file 1 — Supplementary Material [file 10-1055-a-1957-6305-s22060274.pdf]

Supplementary Table S1 Characteristics of all incident SSRI and TCA users at the moment of initiation

General characteristics	Incident SSRI users		Incident TCA users	
Patients	1,182		456	
Treatment periods	1,187		456	
Male (%)	502	(42)	187	41
Age, y (SD)	76	(13)	78	(12)
INR target range (%)				
Low	1,111	(94)	421	(92)
High	76	(6)	35	(8)
Treatment indication (%)				
Atrial fibrillation	880	(74)	357	(78)
Venous thromboembolism	156	(13)	44	(10)
Mechanical heart valves	39	(3)	11	(2)
Vascular disease	40	(3)	20	(4)
Ischemic heart disease	43	(4)	18	(4)
Postoperative	8	(1)	4	(1)
Other	25	(2)	4	(1)
Vitamin K antagonist (%)				
Phenprocoumon	490	(41)	138	(30)
Acenocoumarol	694	(59)	318	(70)
Warfarin	3	(0)	0	(0)
Fluindione	0	(0)	0	(0)
Anticoagulation clinic (%)				
Leiden	539	(45)	162	(36)
Rotterdam	648	(55)	294	(65)
Co-medication (%)				
Antiplatelet drugs	87	(7)	53	(12)
NSAIDs	93	(8)	44	(10)
Proton pump inhibitors	445	(38)	161	(35)

Abbreviations: INR, international normalized ratio; NSAID, nonsteroidal anti-inflammatory drug; SD, standard deviation; SSRI, selective serotonin reuptake inhibitor; TCA, tricyclic antidepressant.

 © 2022. The Author(s).

Supplementary Table S2 Characteristics of incident SSRI and TCA users included in the major bleeding analyses at the moment of initiation

General characteristics	Incident SSRI users		Incident TCA users	
Patients	1,127[a]		390[b]	
Treatment periods	1,132		390	
Male (%)	479	(43)	164	(42)
Age, y (SD)	76	(13)	79	(12)
INR target range (%)				
Low	1,052	(93)	360	(92)
High	75	(7)	30	(8)
Treatment indication (%)				
Atrial fibrillation	828	(74)	308	(79)
Venous thromboembolism	151	(13)	34	(9)
Mechanical heart valves	39	(4)	9	(2)
Vascular disease	38	(3)	19	(5)
Ischemic heart disease	43	(4)	16	(4)
Postoperative	7	(1)	2	(1)
Other	25	(2)	4	(1)
Vitamin K antagonist (%)				
Phenprocoumon	468	(42)	126	(32)
Acenocoumarol	656	(58)	264	(68)
Warfarin	3	(0)	0	(0)
Fluindione	0	(0)	0	(0)
Anticoagulation clinic (%)				
Leiden	515	(46)	150	(39)
Rotterdam	612	(54)	240	(62)
Co-medication (%)				
Antiplatelet drugs	82	(7)	45	(12)
NSAIDs	89	(8)	35	(9)
Proton Pump Inhibitors	423	(38)	141	(36)

Abbreviations: INR, international normalized ratio; NSAID, nonsteroidal anti-inflammatory drug; SD, standard deviation; SSRI, selective serotonin reuptake inhibitor; TCA, tricyclic antidepressant.
[a]Of the 1,182 SSRI initiators, 55 experienced major bleeding before SSRI initiation.
[b]Of the 456 TCA initiators, 26 experienced major bleeding before TCA initiation and 40 were classified as SSRI initiators because of concurrent use.

 © 2022. The Author(s).

Supplementary Table S3 Association of SSRI and TCA initiation with an INR $\geq$ 4 within 2 months after initiation stratified by VKA type

	Total no. of patients	No. of patients with at least 1 INR $\geq$ 4 (%)		OR (95% CI) INR $\geq$ 4
All patients				
Matched non-SSRI users	5,637	1,255	(22)	Reference
All SSRI users	1,175[a]	462	(39)	2.39 (2.08–2.75)
CYP2C9-inhibiting SSRIs	62	26	(42)	2.81 (1.52–5.21)
Non-CYP2C9-inhibiting SSRIs	1,113	436	(39)	2.37 (2.06–2.74)
Matched non-TCA users	2,195	491	(22)	Reference
TCA users	451[a]	150	(33)	1.75 (1.39–2.19)
Acenocoumarol patients				
Matched non-SSRI users	3,341	840	(25)	Reference
All SSRI users	689	316	(46)	2.62 (2.20–3.13)
CYP2C9-inhibiting SSRIs	37	15	(41)	2.78 (1.23–6.28)
Non-CYP2C9-inhibiting SSRIs	652	301	(46)	2.62 (2.18–3.13)
Matched non-TCA users	1,537	381	(25)	Reference
TCA users	313	114	(36)	1.75 (1.34–2.27)
Phenprocoumon patients				
Matched non-SSRI users	2,293	415	(18)	Reference
All SSRI users	484	145	(30)	2.03 (1.61–2.56)
CYP2C9-inhibiting SSRIs	25	11	(44)	2.86 (1.12–7.32)
Non-CYP2C9-inhibiting SSRIs	459	134	(29)	1.98 (1.56–2.52)
Matched non-TCA users	658	110	(17)	Reference
TCA users	138	36	(26)	1.74 (1.10–2.77)

Abbreviations: CI, confidence interval; CYP2C9, cytochrome P450 2C9; INR, international normalized ratio; OR, odds ratio; SSRI, selective serotonin reuptake inhibitor; TCA, tricyclic antidepressant; VKA, vitamin K antagonist.
[a]12 SSRI initiators and 5 TCA initiators were not included in this analysis because no suitable match could be found.

 © 2022. The Author(s).

Supplementary Table S4 Association of SSRI and TCA initiation with dosage of VKAs before and after stratified by VKA type

	No. of patients	Mean dosage before start SSRI/ TCA (tablets)	Mean dosage after start SSRI/ TCA (tablets)	Mean dosage difference (tablets, 95% CI)	Mean dosage difference (percentage, 95% CI)
All patients					
All SSRI users	1,156[a]	1.49	1.42	−0.07 (−0.10 to −0.04)	−3.4 (−4.5 to −2.3)
CYP2C9-inhibiting SSRIs	60	1.78	1.53	−0.24 (−0.47 to −0.02)	−8.6 (−14.2 to −2.9)
Non-CYP2C9-inhibiting SSRIs	1,096	1.47	1.41	−0.06 (−0.09 to −0.03)	−3.1 (−4.2 to −2.0)
TCA users	443[a]	1.62	1.61	−0.01 (−0.05 to 0.02)	−0.2 (−2.5 to 2.1)
Acenocoumarol patients					
All SSRI users	688	2.12	2.02	−0.10 (−0.15 to −0.05)	−2.9 (−4.5 to −1.2)
CYP2C9-inhibiting SSRIs	36	2.49	2.12	−0.38 (−0.75 to 0.00)	−10.8 (−19.9 to −1.8)
Non-CYP2C9-inhibiting SSRIs	652	2.10	2.01	−0.09 (−0.12 to −0.04)	−2.4 (−4.1 to −0.8)
TCA users	311	2.05	2.03	−0.02 (−0.06 to 0.03)	0.5 (−2.7 to 3.6)
Phenprocoumon patients					
All SSRI users	466	0.56	0.54	−0.02 (−0.03 to −0.02)	−4.1 (−5.3 to −2.8)
CYP2C9-inhibiting SSRIs	24	0.70	0.66	−0.05 (−0.09 to −0.01)	−5.2 (−10.0 to −0.3)
Non-CYP2C9-inhibiting SSRIs	442	0.55	0.53	−0.02 (−0.03 to −0.01)	−4.0 (−5.3 to −2.7)
TCA users	132	0.63	0.63	0.00 (−0.02 to 0.01)	−1.6 (−3.9 to 0.7)

Abbreviations: CI, confidence interval; CYP2C9, cytochrome P450 2C9; SSRI, selective serotonin reuptake inhibitor; TCA, tricyclic antidepressant; VKA, vitamin K antagonist.
[a]31 SSRI initiators and 13 TCA initiators were excluded from this analysis because VKA dosages before or after SSRI/TCA initiation were missing.

 © 2022. The Author(s).

Supplementary Table S5 Association of SSRI use with major bleeding stratified by VKA type—crude hazard ratios

	Person-years	No. of cases with major bleeding	HR[a] (95% CI)
All patients			
Nonusers	132,176	2,385[b]	Reference
All SSRI users	4,204	97	1.31 (1.07–1.61)
CYP2C9-inhibiting SSRIs	321	7	1.22 (0.58–2.55)
Non-CYP2C9-inhibiting SSRIs	3,883	90	1.32 (1.07–1.63)
TCA users	1,026	22	1.21 (0.79–1.84)
Acenocoumarol patients			
Nonusers	100,138	1,546	Reference
All SSRI users	2,604	49	1.26 (0.94–1.67)
CYP2C9-inhibiting SSRIs	201	4	1.31 (0.49–3.49)
Non-CYP2C9-inhibiting SSRIs	2,403	45	1.25 (0.93–1.68)
TCA users	676	13	1.25 (0.73–2.16)
Phenprocoumon patients			
Nonusers	31,875	838	Reference
All SSRI users	1,595	48	1.18 (0.88–1.58)
CYP2C9-inhibiting SSRIs	120	3	0.96 (0.31–2.98)
Non-CYP2C9-inhibiting SSRIs	1,475	45	1.20 (0.89–1.62)
TCA users	350	9	1.00 (0.52–1.93)

Abbreviations: CI, confidence interval; CYP2C9, cytochrome P450 2C9; SSRI, selective serotonin reuptake inhibitor; TCA, tricyclic antidepressant; VKA, vitamin K antagonist.
[a]Time-dependent analysis.
[b]One of these major bleedings was observed in a warfarin user.

 © 2022. The Author(s).

Supplementary Table S6 Association of SSRI use with different sites of major bleeding

	Person-years	No. of cases with major bleeding	Incidence rate per 100 person-years (95%CI)	HR[a] (95% CI)	HR[b] (95% CI)
All types of bleeding					
Nonusers	132,176	2,385[c]	1.80 (1.73–1.88)	Reference	Reference
All SSRI users	4,204	97[c]	2.31 (1.88–2.80)	1.31 (1.07–1.61)	1.22 (0.99–1.50)
TCA users	1,026	22[c]	2.14 (1.38–3.19)	1.21 (0.79–1.84)	1.01 (0.66–1.53)
Gastrointestinal bleeding					
Nonusers	132,176	921	0.70 (0.65–0.74)	Reference	Reference
All SSRI users	4,204	37	0.88 (0.63–1.20)	1.30 (0.94–1.81)	1.22 (0.87–1.69)
TCA users	1,026	11	1.07 (0.56–1.86)	1.57 (0.87–2.85)	1.27 (0.70–2.31)
Intracranial bleeding					
Nonusers	132,176	439	0.33 (0.30–0.36)	Reference	Reference
All SSRI users	4,204	23	0.55 (0.36–0.81)	1.64 (1.08–2.49)	1.38 (0.91–2.12)
TCA users	1,026	4	0.39 (0.12–0.94)	1.16 (0.43–3.11)	0.88 (0.33–2.35)
Cutaneous bleeding					
Nonusers	132,176	194	0.15 (0.13–0.17)	Reference	Reference
All SSRI users	4,204	12	0.29 (0.15–0.49)	2.01 (1.12–3.60)	1.85 (1.02–3.34)
TCA users	1,026	1	0.10 (0.00–0.48)	0.66 (0.09–4.73)	0.56 (0.08–4.01)
Joint or muscular bleeding					
Nonusers	132,176	88	0.07 (0.05–0.08)	Reference	Reference
All SSRI users	4,204	3	0.07 (0.02–0.19)	1.08 (0.34–3.41)	0.9 (0.29–2.95)
TCA users	1,026	–	–	–	–
Epistaxis					
Nonusers	132,176	152	0.12 (0.10–0.13)	Reference	Reference
All SSRI users	4,204	6	0.14 (0.06–0.30)	1.31 (0.58–2.96)	1.34 (0.59–3.07)
TCA users	1,026	1	0.10 (0.00–0.48)	0.87 (0.12–6.23)	0.85 (0.12–6.06)
Ocular bleeding					
Nonusers	132,176	25	0.02 (0.01–0.03)	Reference	Reference
All SSRI users	4,204	–	–	–	–
TCA users	1,026	–	–	–	–
Urinary tract bleeding					
Nonusers	132,176	304	0.23 (0.21–0.26)	Reference	Reference
All SSRI users	4,204	9	0.21 (0.10–0.39)	0.96 (0.50–1.87)	1.08 (0.55–2.10)
TCA users	1,026	3	0.29 (0.07–0.80)	1.31 (0.42–4.08)	1.27 (0.41–3.97)
Traumatic bleeding					
Nonusers	132,176	10	0.01 (0.00–0.01)	Reference	Reference
All SSRI users	4,204	–	–	–	–
TCA users	1,026	–	–	–	–
Respiratory tract bleeding					
Nonusers	132,176	116	0.08 (0.07–0.10)	Reference	Reference
All SSRI users	4,204	2	0.05 (0.01–0.15)	0.57 (0.14–2.29)	0.51 (0.12–2.07)
TCA users	1,026	–	–	–	–
Other sites of major bleeding[d]					
Nonusers	132,176	150	0.11 (0.10–0.13)	Reference	Reference

 © 2022. The Author(s).

Supplementary Table S6 (*Continued*)

	Person-years	No. of cases with major bleeding	Incidence rate per 100 person-years (95%CI)	HR[a] (95% CI)	HR[b] (95% CI)
All SSRI users	4,204	5	0.12 (0.04–0.26)	1.09 (0.45–2.66)	0.98 (0.40–2.41)
TCA users	1,026	3	0.29 (0.07–0.80)	2.64 (0.84–8.28)	2.21 (0.70–6.96)

Abbreviations: CI, confidence interval; HR, hazard ratio; SSRI, selective serotonin reuptake inhibitor; TCA, tricyclic antidepressant.
[a]Time-dependent analysis.
[b]Time-dependent analysis adjusted for sex and time-dependent covariates age, INR target range, indication for VKA, and co-medication (antiplatelet drugs, NSAIDs, and PPIs).
[c]The sum of the different major bleeding classes exceeds the number of major bleeding since some bleedings are classified in two categories.
[d]Including bleeding of circulatory system, retroperitoneal bleeding, and other types of bleeding.

Supplementary Table S7 Association of SSRI use with major bleeding for incident and prevalent users stratified by VKA type—crude hazard ratios

	Person-years	No. of major bleedings	HR[a] (95% CI)
All patients			
Nonusers	132,176	2,385[b]	Reference
Incident SSRI users	1,837	35	1.22 (0.87–1.70)
Prevalent SSRI users	2,367	62	1.38 (1.07–1.77)
Incident TCA users	528	12	1.44 (0.81–2.53)
Prevalent TCA users	498	10	1.02 (0.55–1.89)
Acenocoumarol patients			
Nonusers	100,138	1,546	Reference
Incident SSRI users	1,157	22	1.42 (0.93–2.17)
Prevalent SSRI users	1447	27	1.14 (0.78–1.68)
Incident TCA users	329	8	1.77 (0.88–3.55)
Prevalent TCA users	348	5	0.85 (0.36–2.06)
Phenprocoumon patients			
Nonusers	31,875	838	Reference
Incident SSRI users	677	13	0.82 (0.48–1.43)
Prevalent SSRI users	918	35	1.41 (1.00–1.97)
Incident TCA users	199	4	0.86 (0.32–2.29)
Prevalent TCA users	151	5	1.16 (0.48–2.80)

Abbreviations: CI, confidence interval; HR, hazard ratio; SSRI, selective serotonin reuptake inhibitor; TCA, tricyclic antidepressant; VKA, vitamin K antagonist.
[a]Time-dependent analysis.
[b]One of these major bleedings was observed in a warfarin user.

 © 2022. The Author(s).
